# Chronic Resistance Exercise Combined with Nutrient Timing Enhances Skeletal Muscle Mass and Strength While Modulating Small Extracellular Vesicle miRNA Profiles

**DOI:** 10.3390/biomedicines14010127

**Published:** 2026-01-08

**Authors:** Dávid Csala, Zoltán Ádám, Zoltán Horváth-Szalai, Balázs Sebesi, Kitti Garai, Krisztián Kvell, Márta Wilhelm

**Affiliations:** 1 Doctoral School of Biology and Sports Biology, Faculty of Sciences, University of Pécs, Ifjúság Str. 6, 7624 Pécs, Hungary; csaladav@gamma.ttk.pte.hu; 2 Department of Pharmaceutical Biotechnology, Faculty of Pharmacy, University of Pécs, Rókus Str. 2, 7624 Pécs, Hungary; garai.kitti@pte.hu (K.G.); kvell.krisztian@pte.hu (K.K.); 3 Department of Laboratory Medicine, Medial School, University of Pécs, Ifjúság Str. 13, 7624 Pécs, Hungary; horvath-szalai.zoltan@pte.hu; 4 Department of Sports Biology and Kinesiology, Institute of Sports Science and Physical Education, Faculty of Science, University of Pécs, Ifjúság Str. 6, 7624 Pécs, Hungary; sebesi.balazs@pte.hu; 5 Department of Leisure Sports and Recreation, Institute of Sports Science and Physical Education, Faculty of Science, University of Pécs, Ifjúság Str. 6, 7624 Pécs, Hungary; mwilhelm@gamma.ttk.pte.hu

**Keywords:** anabolic window, hypertrophy, exosome, protein synthesis, PTEN, body composition, resistance training, adaptation

## Abstract

**Background**: The anabolic window hypothesis suggests a limited post-exercise period for optimal nutrient uptake and utilization. Prior research indicates that miRNAs in extracellular vesicles (EVs) may regulate post-exercise adaptation by influencing protein synthesis. This study aimed to examine the effects of resistance exercise (RE) on physiological parameters and the expression and function of miRNAs transported in EVs. **Methods**: Twenty resistance-trained male participants (22 ± 2 years) completed a five-week RE program designed for hypertrophy. They consumed maltodextrin and whey protein based on assigned nutrient timing: immediately post-exercise (AE), three hours post-exercise (AE3), or no intake (CTRL). Body composition and knee extensor strength were assessed. Small EVs were isolated and then validated via three methods. Nanoparticle tracking analysis determined EV concentration and size, followed by pooled miRNA profiling and signaling pathway analysis. **Results**: Skeletal muscle mass significantly increased in AE (*p* = 0.001, g = 2) and AE3 (*p* = 0.028, g = 1), and it was higher in AE compared to CTRL (*p* = 0.013, η^2^ = 0.41), while knee extensor strength improved only in AE (*p* = 0.032, g = 0.9). Body fat percentage significantly decreased in all groups, AE (*p* = 0.005, g = 1.5), AE3 (*p* = 0.024, g = 1), and CTRL (*p* = 0.005, g = 1.7). Vesicle concentration significantly increased in the AE group (*p* = 0.043, r = 0.7), while it decreased in the CTRL group (*p* = 0.046, r = 0.8). Distinct miRNA expression profiles emerged post-intervention: 20 miRNAs were upregulated in AE, while 13 in AE3 and 15 in CTRL were downregulated. **Conclusions**: Nutrient timing influences training adaptation but is not more critical than total macronutrient intake. Changes in EV-transported miRNAs may regulate anabolic processes via the PI3K-AKT-mTOR and FoxO pathways through PTEN regulation.

## 1. Introduction

According to the anabolic window theory, nutrients consumed immediately after exercise are utilized more effectively than those ingested outside this period [1]. Despite its widespread acceptance, current research has not reached a consensus on the validity or practical relevance of this concept. The lack of agreement is largely attributable to heterogeneous study designs, methodological limitations, and pronounced inter-individual variability in responses to standardized exercise and nutrition protocols. Nevertheless, post-exercise nutrition remains a central element of sports nutrition practice and is heavily promoted by dietary supplement companies, particularly in the form of post-workout protein shakes, raising the question of whether such strategies truly enhance muscle hypertrophy.

Resistance exercise (RE) consists of repeated muscle contractions against external loads and is widely applied to increase or maintain skeletal muscle (SM) mass and strength, especially when combined with appropriate nutritional intake [2]. Adaptive responses of SM occur predominantly during the post-exercise recovery period and are strongly influenced by training load and intensity [3]. The immediate post-exercise period is considered a critical phase for nutrient timing, as carbohydrate and protein ingestion can support glycogen replenishment, stimulate protein synthesis, and attenuate muscle protein breakdown [4]. At the cellular level, nutrient availability and mechanical loading interact to regulate skeletal muscle mass through multiple signaling pathways [5,6,7].

Among these pathways, the phosphoinositide 3-kinase (PI3K)–AKT–mechanistic target of rapamycin (mTOR) signaling cascade plays a pivotal role in resistance exercise-induced muscle hypertrophy [8]. Activation of AKT leads to downstream stimulation of mTOR, thereby promoting protein synthesis and muscle growth [9,10]. While the involvement of this pathway is well established, several molecular sensors and regulatory mechanisms contributing to phenotypic adaptations following RE remain only partly understood [11].

MicroRNAs (miRNAs) are single-stranded, non-coding RNA molecules whose primary function is to inhibit gene expression at the post-transcriptional level by targeting mRNAs [12]. These molecules fine-tune protein synthesis in the body, a process essential for post-exercise adaptations. Circulating miRNAs are transported in the bloodstream bound to proteins such as Argonaute, associated with high-density lipoproteins, or encapsulated within extracellular vesicles (EVs) [13]. EVs are released by most cell types and facilitate intercellular communication by transferring miRNAs and other bioactive molecules between tissues [14]. Growing evidence indicates that physical activity stimulates the release of EVs from SM and other tissues into the circulation, suggesting an important role for EV-mediated signaling in systemic exercise adaptations [15].

The effects of RE on EV and miRNA profiles have been increasingly investigated, with evidence suggesting that RE may differentially influence these molecular responses [16]. However, most studies published between 2018 and 2023 investigating the miRNA profile in serum or plasma in response to chronic RE primarily focused on aging populations [17,18,19,20,21,22,23,24,25], with only one study specifically examining young males [26]. Many studies included mixed-sex cohorts, focused on clinical populations, or combined RE with additional interventions, further complicating the interpretation and comparison of findings. Acute changes in EV-associated miRNAs following blood flow-restricted RE have been reported in young men [27], while only one study has investigated chronic resistance training-induced alterations in EV-derived miRNAs, comparing older adults undertaking a home-based training program with younger non-training controls [28]. To date, no study examined the combined effects of RE and nutrient timing on EV-associated miRNA profiles.

Therefore, the global aim of the present study is to investigate the effects of resistance exercise combined with nutrient timing on physiological and molecular adaptations associated with muscle hypertrophy. Specifically, we aimed to (i) assess changes in body composition and muscle strength following a 5-week hypertrophy-oriented resistance training program; (ii) examine molecular markers related to protein synthesis, with a particular focus on extracellular vesicle-associated miRNAs; and (iii) analyze alterations in circulating EV concentration and their miRNA profiles in response to the timing of carbohydrate–protein complex ingestion relative to resistance exercise.

## 2. Materials and Methods

### 2.1. Study Protocol and Patient Enrolment

The study was conducted in accordance with the Declaration of Helsinki and was approved by the National Public Health Center (NNGYK) with the professional and ethical approval of the Scientific and Research Ethics Committee of the Health Science Council (ETT TUKEB), Hungary (approval number NNGYK/30340-6/2025, ethical opinion BM/13764-1/2025).

Twenty healthy young male volunteers (age: 22 ± 2 years) with at least one year of resistance training experience and currently training a minimum of three times per week were recruited for the study. Participants were in good general health, defined as the absence of any acute or chronic medical conditions (e.g., metabolic disorders, cardiovascular diseases, cancer). Exclusion criteria included smoking, alcohol consumption, medication use, engagement in any other regular sport activity, and intake of dietary supplements within two months preceding the study. Prior to participation, all subjects provided written informed consent, confirming their willingness to comply with the study protocol and acknowledging that they had received both verbal and written explanations of the study procedures.

### 2.2. Blood Sampling and PFP Isolation

Before the start of the 5-week study period, participants arrived at the sampling site in the morning. They were instructed to refrain from significant physical activity for two days prior to sampling. Following a 30 min rest period, fasting blood samples were collected. Venous blood was drawn from the antecubital vein using a closed blood collection system into EDTA and citrate tubes (BD Vacutainer^®^, Franklin Lakes, NJ, USA) by trained nursing personnel under medical supervision. EDTA-anticoagulated whole blood samples were analyzed for complete blood count using a Sysmex XN hematology analyzer at the Department of Laboratory Medicine, University of Pécs, Hungary (accreditation No. NAH-9-0008/2021). Heart rate and blood pressure were also measured. Platelet-free plasma (PFP) was subsequently isolated from citrate-anticoagulated blood samples to prevent ex vivo vesicle release [29]. After isolating PFP, samples were frozen at −80 °C.

### 2.3. Body Composition and Muscle Strength Assessment

Body composition was assessed using an InBody 770 bio-impedance analyzer (InBody Co., Ltd., Seoul, Republic of Korea). Following a standardized warm-up, the maximum strength of the knee extensors was measured using a computer-assisted dynamometer (Multicont II, Mediagnost, Budapest, and Mechatronic Kft, Szeged, Hungary). The quadriceps femoris is the largest muscle group in the body [30], making it an excellent indicator of individual strength capacity. The torque produced by the knee extensors was measured using the device with a Newton meter (Nm) precision.

### 2.4. Diet Composition

Following sample collection, participants were randomly assigned to one of three groups: AE, consuming a carbohydrate–protein supplement immediately post-exercise; AE3, consuming the same supplement 3 h post-exercise; and CTRL, receiving no supplementation. An isocaloric placebo was not used to avoid potential metabolic interference with training adaptations. Participants then completed the 5-week training program while consuming fast-digesting carbohydrates (unflavored GymBeam maltodextrin) and whey protein (Nutriversum Pure Whey Pro, chocolate flavor). Daily carbohydrate and protein intake was standardized at 2.2 g/kg body weight, consistent with previous recommendations and studies [31,32]. The combined post-exercise intake of carbohydrates and protein was selected based on evidence showing a greater insulin response compared with carbohydrate intake alone [33], which is relevant for muscle recovery and anti-catabolic processes in resistance-trained individuals [1]. Supplementation was provided only on training days and consumed post-exercise according to group allocation, with per-meal intake calculated by dividing total daily intake into five portions.

### 2.5. Exercise Protocol and Study Group

Participants completed a 5-week supervised resistance training program (4 sessions/week) based on progressive overload, with increasing training volume aimed at inducing muscle hypertrophy. The program included both compound (e.g., deadlifts, bench press, squats; 6–12 repetitions, 90–150 s rest) and isolation exercises (e.g., dumbbell flies, stiff-arm pushdowns; 10–15 repetitions, 45–60 s rest). Training protocols were varied weekly to provide novel stimuli and promote continuous adaptation. One-repetition maximum (1RM) was not assessed; instead, participants performed working sets to volitional fatigue under personal trainer supervision. For further details of the training program, see Supplementary Material S1. At the end of the intervention, fasting blood samples were collected, and knee extensor strength and body composition were reassessed following a two-day rest period, identical to baseline conditions. To avoid acute supplementation effects, the final sampling occurred at least 48 h after the last supplement intake.

### 2.6. Isolation of EVs

Before starting the isolation protocol, samples were pooled for miRNA profiling to capture representative patterns across participants and facilitate subsequent analyses. For the AE and AE3 groups, 100 µL of PFP was pooled from each participant, while in the CTRL group, 120 µL of PFP per individual was pooled. Ultimately, 700 µL of PFP was utilized as the starting volume for each group. For TEM and protein array analysis, EVs were isolated from a randomly selected PFP sample. For NTA measurements, the starting volume was also 700 µL of PFP per individual. During EV isolation, samples were filtered through an 800 nm filter and centrifuged at 20,500× *g*, pelleting larger EVs. These were resuspended in PBS and stored at −80 °C for later use. In this study, only small EVs were analyzed; larger EVs were separated during the isolation process but were not further examined. From the supernatant, small EVs were isolated using the Total Exosome Isolation (from plasma) Kit (Invitrogen, Thermo Fisher Scientific, Waltham, MA, USA) according to the manufacturer’s instructions. Samples intended for TEM, NTA, and TaqMan^®^ Advanced miRNA Assay analyses were processed with protein digestion during isolation, while samples prepared for Exo-Check Protein Array analysis were processed without digestion.

### 2.7. Nanoparticle Tracking Analysis (NTA)

Preparations containing small EVs were analyzed and quantified using the Nanosight NS300 instrument (Malvern Panalytical Ltd., Malvern, UK). The camera level was manually adjusted for each sample to optimize particle visualization in accordance with the manufacturer’s guidelines. Samples were introduced using a syringe pump (infusion rate = 50). The detection threshold was set to maximize sensitivity while minimizing background noise. Each sample was measured in five replicates, with 60 s videos captured for each replicate. The recorded videos were then processed and analyzed using the built-in NTA v3.2 software [34]. All samples were diluted in PBS, with optimal measurement concentrations determined through preliminary testing to achieve the ideal particle count per frame (40–100 particles/frame) [35].

### 2.8. Transmission Electron Microscopy (TEM)

Small EVs were visualized using TEM. A mixture of uranyl acetate (5%) and absolute alcohol (98%) in a ratio of 1:1 was aspirated using a syringe and immediately wrapped in aluminum foil. Six drops of uranyl acetate and 3 × 6 drops of water were placed on a grid holder plate, and then the grids were incubated for 15 min. The same procedure was followed for lead citrate, and after 3 min, the grids were washed by placing them on water droplets. Excess liquid was removed from the grids, and then they were carefully transferred to the grid holder after draining properly. After drying overnight, grids were examined using TEM and then with a JEOL TEM 1200 EX (JEOL Ltd., Tokyo, Japan). The average diameter of the isolated EVs was measured using three independent TEM preparations and analyzed with ImageJ software (National Institutes of Health, Bethesda, MD, USA).

### 2.9. Exo-Check Protein Array

The Exo-Check™ Exosome Antibody Array was used to validate the efficiency of vesicle isolation, enabling the simultaneous identification of eight surface exosomal protein markers. Briefly, the Exo-Check™ Protein Array protocol began with the resuspension of EVs in 1× PBS and the quantification of the protein concentration using Pierce™ BCA Protein Assay. From this, 50 µg of protein was used, mixed with a 10× Lysis Buffer to achieve a 1× final concentration, and then briefly vortexed. A kabeling reagent was added, and the sample was incubated for 30 min at room temperature. After incubation, the excess labeling reagent was removed using purification columns. The labeled EV lysate was combined with the Blocking Buffer and applied to the membrane, and it was then incubated overnight at 2–8 °C with gentle shaking. The next day, the membrane was washed, treated with the Detection Buffer, and the signals were visualized according to the manufacturer’s recommendations.

### 2.10. Small EV Total RNA Purification and miRNA Profiling

Total RNA from small EVs was extracted using the Total Exosome RNA and Protein Isolation Kit (Invitrogen, Thermo Fisher Scientific, Waltham, MA, USA) according to the manufacturer’s protocol. miRNA quantification was performed using the TaqMan^®^ Advanced miRNA Assay following the manufacturer’s protocol. First, total RNA was reverse-transcribed into cDNA using the TaqMan^®^ Advanced miRNA cDNA Synthesis Kit, which includes poly(A) tailing and adapter ligation steps to ensure efficient amplification. Next, the cDNA was amplified with specific TaqMan^®^ primers designed for each target miRNA. We examined the expression of 380 miRNAs isolated from EVs and their potential roles in physiological processes under mechanical loading (pre–post) combined with nutrient timing. Real-time PCR was conducted using the TaqMan array cards, enabling the simultaneous detection of miRNAs in 384 wells (TaqMan^®^ Advanced miRNA Assay Thermo Fisher Scientific, Waltham, MA, USA; QuantStudio™ 12K Flex Real-Time PCR System, Thermo Fisher Scientific, Waltham, MA, USA). The results were analyzed, determining miRNA expression levels relative to pre-exercise data (ExpressionSuite release v1.3 software, Thermo Fisher Scientific, Waltham, MA, USA). During data analysis, the expression levels of individual miRNAs were determined using the delta–delta Ct (∆∆Ct) method, with global normalization applied. To verify the quality of data, statistical parameters provided by the software were utilized (e.g., amplification efficiency, distribution of Ct values). The following flag settings were applied to ensure reliable miRNA expression determination. Wells with an amplification score below 1, a Cq confidence below 0.8, and an amplification algorithm result = 0.3 were excluded from the analysis. Additionally, miRNAs with Ct values above 32 were excluded, as this value is still considered reliable according to the Thermo Fisher user manual. miRNAs were considered to have significantly different expression if their RQ values fell below 0.5 or above 2 in the group comparison. Subsequently, the pooled, normalized relative expression values were represented in a Venn diagram.

### 2.11. miRNA Target Prediction and Pathway Analysis

After identifying several differentially expressed miRNAs in the AE, AE3, and CTRL groups, miRNA-mRNA signaling pathway interaction analysis was performed. For this, the DIANA-miRPath v4.0 bioinformatics software was utilized, which is available online [36]. The identification of target genes for each miRNA was based on the miRTarBase 2022 database, considering only experimentally validated interactions with a “strong” rating. If no significant miRNA–target gene interaction was found for a particular miRNA, no secondary target annotation source was used. Analyses were conducted using homo sapien species-specific data, and the miRNA annotation was obtained from the miRBase v22.1 database. The identification of relevant biological pathways was based on the KEGG (Kyoto Encyclopedia of Genes and Genomes) database. During data analysis, target gene unification was performed using the “genes union” method. For statistical evaluation, classical analysis was applied, and the significance threshold was set at *p* < 0.05. To control the false discovery rate (FDR), FDR correction was applied. Post-enrichment analysis was not permitted, and long non-coding RNA targets were excluded from the analysis. In addition, Ingenuity Pathway Analysis (IPA, QIAGEN Inc., Hilden, Germany) was used to complement the DIANA-miRPath results and provide further insight into potential cellular effects of differentially expressed miRNAs. The miRNA Target Filter module was applied to identify experimentally validated mRNA targets of the selected miRNAs, based on findings from TarBase, miRecords, and the Ingenuity Knowledge Base. This approach enabled the integration of published evidence with our dataset to highlight high-confidence miRNA–mRNA interactions. The predicted targets were subsequently analyzed to infer affected cellular functions and regulatory networks. Only experimentally validated interactions were included in the interpretation to increase biological relevance.

### 2.12. Statistics

**Figure 1:**
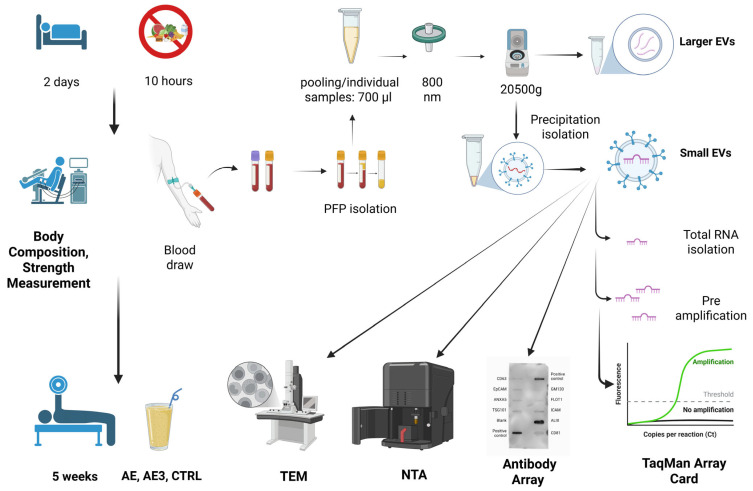
Flowchart representing the entire workflow in the study. AE, consumed nutritional supplements immediately after exercise; AE3, consumed nutritional supplements three hours after exercise; CTRL, did not consume any nutritional supplements; TEM, transmission electron microscopy; NTA, nanoparticle tracking analysis; PFP, platelet-free plasma. Created in BioRender. Csala, D. (2025). https://BioRender.com/gqa51jk.

Statistical calculations were performed using SPSS software (IBM SPSS Statistics 25) and Excel. Descriptive statistical methods were used to examine the distribution. Due to the sample size, the Shapiro–Wilk normality test was applied. Following the normality test, paired *t*-tests or Wilcoxon tests were used to compare pre- and post-exercise values. Random selection was applied, resulting in differences between groups; therefore, changes relative to themselves (pre, post) were analyzed. Baseline differences between groups were assessed using one-way analysis of variance (ANOVA) for demographic, body composition, and strength variables, and analysis of covariance (ANCOVA) was employed to compare changes in skeletal muscle mass and knee extensor strength between groups, with pre-exercise values included as covariates. Statistical significance was set at *p* < 0.05. The effect size for the paired *t*-test was estimated using Hedges’ g. The interpretation of effect size values was based on the following thresholds: values between 0.00 and 0.20 were considered very weak, 0.20 to 0.50 weak, 0.50 to 0.80 moderate, 0.80 to 1.20 strong, 1.20 to 2.00 very strong, and values exceeding 2.00 were regarded as indicating extremely strong effects. In instances when the Wilcoxon signed-rank test was employed, effect size was calculated using Cohen’s r. The interpretation of r followed a similar threshold-based approach: values from 0.00 to 0.20 were classified as very low, 0.20 to 0.40 as low, 0.40 to 0.60 as moderate, 0.60 to 0.80 as strong, and values between 0.80 and 1.00 as very strong. To examine between-group differences following intervention, ANCOVA was conducted, with pre-intervention scores included as covariates. Prior to performing ANCOVA, all necessary assumptions for the test were assessed and confirmed. In case a statistically significant main effect of group was identified, Bonferroni-adjusted pairwise comparisons based on estimated marginal means were carried out, determining specific differences between groups. Evaluating the magnitude of observed effects, partial eta squared (η^2^) values were calculated. Effect sizes were interpreted according to the following thresholds: 0.01 indicated a small effect, 0.06 a medium effect, and 0.14 a large effect. Post hoc statistical power (1 − β) was calculated using G*Power software (version 3.1.9.7) based on the observed effect size and the achieved sample size. For easier understanding, the key steps of the entire workflow are summarized in Figure 1.

## 3. Results

### 3.1. Participant Characteristics

The main characteristics of the participants are presented in Table 1. A total of 20 participants were included in the study and allocated into three groups: AE (*n* = 7), AE3 (*n* = 7), and CTRL (*n* = 6). No significant baseline differences were observed between groups for demographic or body composition variables; however, a significant between-group difference was detected in baseline knee extensor strength (*p* = 0.021). Accordingly, pre-exercise values were included as covariates in subsequent analyses.

### 3.2. Results of Body Composition Changes

**Figure 2:**
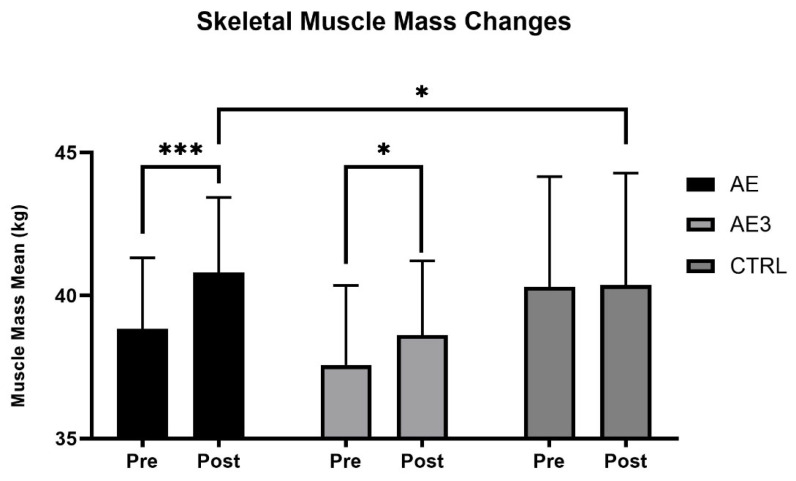
The change in skeletal muscle mass (kg) of three groups: AE, consumed nutritional supplements immediately after exercise; AE3, consumed nutritional supplements three hours after exercise; CTRL, did not consume any nutritional supplements at baseline (pre) and after 5 weeks of load (post). * *p* < 0.05 and *** *p* < 0.001 for pre–post.

According to data measured by the bio-impedance analyzer, the average SM mass increased from 38.8 kg to 40.8 kg (*p* = 0.001, g = 2, 1 − β = 0.99) in the AE group, from 37.5 kg to 38.6 kg (*p* = 0.028, g = 1, 1 − β = 0.76) in the AE3 group, and from 40.3 kg to 40.36 kg (*p* = 0.917) in the CTRL group, in which no significant change was detected. After controlling for baseline values, skeletal muscle mass was significantly higher in the AE group compared to the CTRL group (*p* = 0.013, η^2^ = 0.41, 1 − β = 0.86). No significant differences were observed between the AE and AE3 groups (*p* = 0.24) or between the AE3 and CTRL groups (*p* = 0.47). Changes in SM mass are shown in Figure 2. These findings indicate that SM mass increase was more pronounced in the group consuming the supplement immediately after exercise compared to the group consuming it three hours later or not at all, as supported by the effect size and power analysis. In the AE group, Hedges’ g was 2, indicating a very large effect, and the post hoc power was 0.99, suggesting a highly reliable and statistically robust result. This is further supported by the higher skeletal muscle mass observed in the AE group compared to the CTRL group.

**Figure 3:**
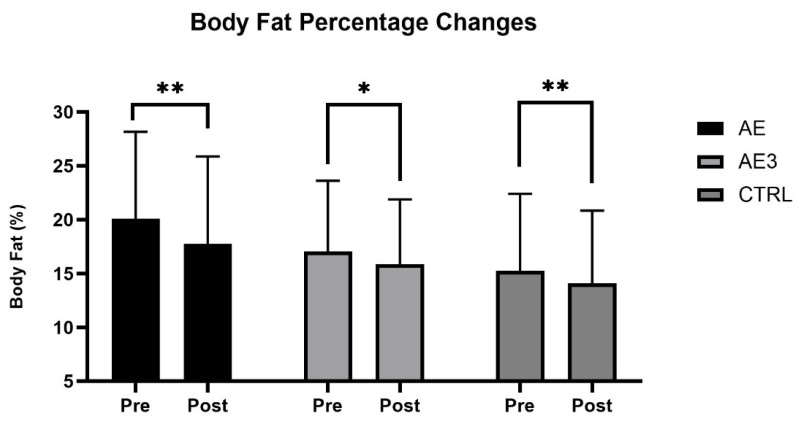
The change in body fat percentage (%) of three groups: AE, consumed nutritional supplements immediately after exercise; AE3, consumed nutritional supplements three hours after exercise; CTRL, did not consume any nutritional supplements at baseline (pre) and after 5 weeks of load (post). * *p* < 0.05 and ** *p* < 0.01 for pre–post.

Body fat percentage decreased in all three groups. In the AE group a reduction was detected from 20% to 17.7% (*p* = 0.005, g = 1.5, 1 − β = 0.97), in the AE3 group from 17% to 15.8% (*p* = 0.024, g = 1, 1 − β = 0.79), and in the CTRL group from 15.2% to 14.1% (*p* = 0.005, g = 1.7, 1 − β = 0.98), as shown in Figure 3. These results suggest that the increase in SM mass over the 5-week period was not due to an unusually high intake of nutrients but rather to the timing of the carbohydrate–protein complex consumption. Strong effect sizes (g = 1.0–1.7) and high post hoc power values (0.79–0.98) across all groups further support the reliability and robustness of these findings.

It is worth noting that all three groups improved body composition over the 5-week period, as body fat percentage decreased in all groups (AE, AE3, CTRL). Simultaneously, SM mass increased in two groups (AE, AE3), while remaining unchanged in the CTRL group, resulting in an improved muscle-to-fat ratio for all groups, demonstrating the effectiveness of the 5-week training program, with more pronounced results observed in the AE and AE3 groups.

Interestingly, SM hypertrophy occurred in the AE and AE3 groups without a mass-gaining diet, while body fat percentage decreased during this period, suggesting that the training program, which included sets performed to muscle exhaustion, was effective for trained individuals, even without using pre-determined sets and repetitions based on 1RM. Instead, weights were adjusted by personal trainers, matching the current energy levels of participants, maximizing performance.

### 3.3. Strength Changes of the Knee Extensors

**Figure 4:**
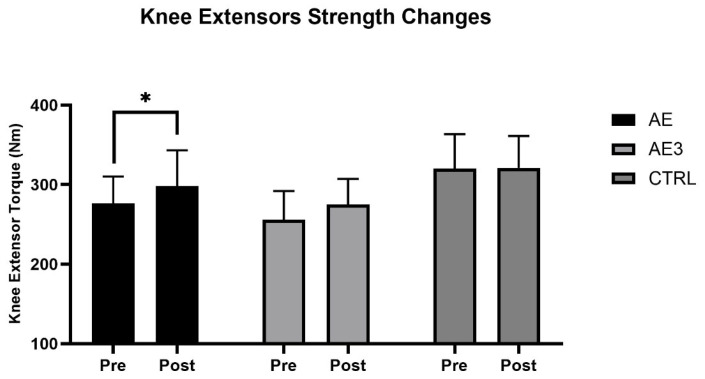
The change in knee extensor torque (Nm) of three groups: AE, consumed nutritional supplements immediately after exercise; AE3, consumed nutritional supplements three hours after exercise; CTRL, did not consume any nutritional supplements at baseline (pre) and after 5 weeks of load (post). * *p* < 0.05 for pre–post.

The average strength of knee extensors in the AE group increased from 276.4 Nm to 296.9 Nm over the 5-week period (*p* = 0.032, g = 0.9, 1 − β = 0.74) (Figure 4), while in the AE3 group, it went from 256.2 Nm to 274.8 Nm, showing an indicative improvement (*p* = 0.054, g = 0.8, 1 − β = 0.63). Although the result was not statistically significant, the effect size indicated a substantial difference in the AE3 group. In the CTRL group, it did not change significantly, moving from 320.3 Nm to 321.1 Nm (*p* = 0.885). No significant differences were found between groups in the knee extensor strength (*p* = 0.34).

### 3.4. Validation of Isolation

**Figure 5:**
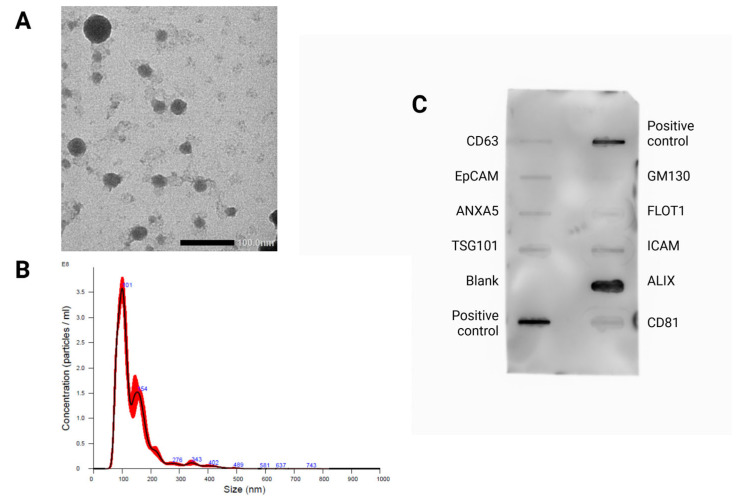
Characterization of small EV-enriched preparations isolated from human blood plasma. (**A**) Transmission electron microscope (TEM) image of small EVs purified from plasma samples (scale bar: 100 nm). (**B**) Analysis of EV size distribution and concentration in small EV-enriched preparations using a NanoSight NS300 instrument. (**C**) Evaluation of exosome-specific protein expression abundance in small EV-enriched preparations using Exo-Check™ Exosome Antibody Array. Created in BioRender. Csala, D. (2025). https://BioRender.com/gqa51jk.

According to the Minimal Information for Studies of Extracellular Vesicles 2018 and 2023 guidelines, at least two distinct validation methods should be employed to identify EVs and confirm their characteristics [37,38]. The validation of small EVs is shown in Figure 5.

### 3.5. Results of the NTA Measurements

**Figure 6:**
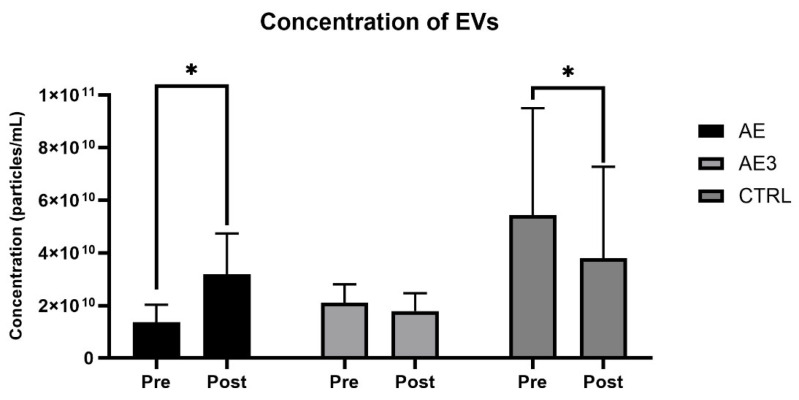
The change in concentration of EVs (particles/mL) in the three studied groups (AE, AE3, CTRL) at baseline (pre) and after 5 weeks of load (post). * *p* < 0.05 for pre–post.

PFP plasma samples were collected in the morning after an overnight fast, following two days of rest, both before and after the 5-week training program. Small EV concentration increased significantly in the AE group, from 1.37 × 1010 to 3.19 × 1010 (*p* = 0.043, r = 0.7, 1 − β = 0.53). In the AE3 group, there was an indicative change from 2.79 × 1010 to 1.79 × 1010 (*p* = 0.5). However, in the CTRL group, small EV concentration decreased significantly from 5.43 × 1010 to 3.81 × 1010 (*p* = 0.046, r = 0.8, 1 − β = 0.5) (Figure 6).

**Figure 7:**
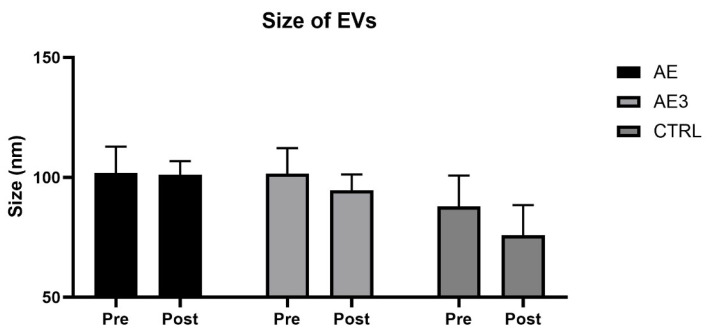
The change in EV modal size (nm) of the three studied groups (AE, AE3, CTRL) at baseline (pre) and after 5 weeks of load (post).

The modal size of small EVs in the AE group changed from 101.8 nm to 101.1 nm (*p* = 0.896), and in the AE3 group, it changed from 101.6 nm to 94.6 nm (*p* = 0.142); however, these changes were not significant. In the CTRL group, a close to significant decrease was found in the modal small EV size from 87.9 nm to 75.8 nm (*p* = 0.056) (Figure 7).

Data indicate that the isolation of small EVs was successful based on particle size, since the modal size of the isolated particles fell within the small EV size range [39]. Interestingly, vesicle concentration increased only in the group showing the most anabolic physiological changes (AE), while in the least anabolic group (CTRL), a reduction in concentration was experienced. Significant changes in vesicle concentration were observed in the AE and CTRL groups, with large effect sizes (r = 0.7–0.8). However, due to the low statistical power, it is advisable to confirm these results in future studies with larger sample sizes.

### 3.6. miRNA Expression Profile Following 5-Week RE

**Figure 8:**
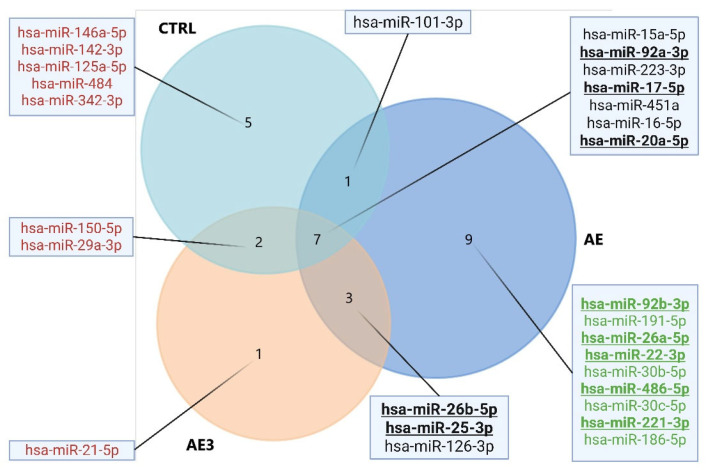
The change in miRNA profile at baseline (pre) and after 5 weeks of load (post), examining three groups (AE, AE3, CTRL). MiRNAs marked in red showed negative expression, those marked in green showed positive expression, and the ones marked in black were negatively expressed in the AE3 and CTRL groups but positively expressed in the AE group. Underlined miRNAs target PTEN. Created in BioRender. Csala, D. (2025). https://BioRender.com/gqa51jk.

The presented data correspond to miRNAs isolated from circulating small EVs. In the AE group, 20 miRNAs were differentially expressed compared to baseline, each with an RQ value greater than 2, while in the AE3 group, 13 miRNAs were differentially expressed, and in the CTRL group, 15 miRNAs were differentially expressed, showing differential expression with an RQ value below 0.5. These differentially expressed miRNAs were depicted in a Venn diagram (Figure 8).

Following the exercise intervention, many changes in miRNAs were detected. The most deregulated were hsa-miR-17-5p, which increased by approximately 3.4-fold in the AE group, while it decreased by approximately 0.4-fold in the CTRL group and 0.3-fold in the AE3 group. hsa-miR-20a-5p increased by approximately 4.7-fold in the AE group, while it decreased by approximately 0.3-fold in the CTRL group and 0.2-fold in the AE3 group. hsa-miR-92a-3p increased by approximately 3.8-fold in the AE group, while it decreased by approximately 0.4-fold in both the CTRL and AE3 groups. Similarly, hsa-miR-92b-3p, sharing the same seed sequence, increased by approximately 4.6-fold in the AE group, but it showed no significant change in the other two groups. These miRNAs are members of the miR-17-92 cluster and regulate similar cellular processes (21). Additionally, in the AE group, hsa-miR-22-3p increased by approximately 2.6-fold, hsa-miR-26a-5p by approximately 4-fold, hsa-miR-221-3p by approximately 4-fold, and hsa-miR-486-5p (myomiR) by approximately 2.5-fold. In AE, hsa-miR-25-3p increased by approximately 2.7-fold, and hsa-miR-26b-5p increased by approximately 3.5-fold, while in AE3, both miRNAs decreased by approximately 0.4-fold. The remaining differentially expressed miRNAs and their RQ values are provided in Supplementary Material Table S1.

### 3.7. Pathway Analysis

**Figure 9:**
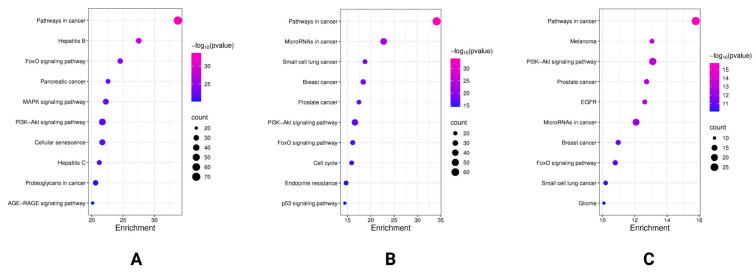
The bubble plots present the top 10 biological pathways targeted by the differentially expressed miRNAs. The color intensity of each bubble indicates the level of statistical significance (with more intense colors representing higher significance), while the bubble size reflects the number of miRNA targets associated with each pathway, in AE, consumed nutritional supplements immediately after exercise; AE3, consumed nutritional supplements three hours after exercise; and CTRL, did not consume any nutritional supplements. Groups intersection (**A**), AE group (**B**), AE and AE3 groups intersection (**C**).

To better understand the role of differentially expressed miRNAs in anabolic processes, we investigated the differentially expressed miRNAs in the most anabolic AE group using DIANA-miRPath v4.0 bioinformatics software. To identify the most relevant targeted pathways for each miRNA, KEGG database analysis was performed. The differentially expressed miRNAs in the AE, AE3, and CTRL groups were involved in two key pathways among the top 10 most significantly altered ones in the regulation of anabolic processes (Figure 9A). The forkhead box O (FoxO) signaling pathway ranked third, with 34 targets (*p* = 2.87 × 10^−25^, FDR: 3.26 × 10^−23^), and the PI3K-AKT one ranked sixth, with 48 targets (*p* = 1.97 × 10^−22^, FDR: 9.90 × 10^−21^). All seven miRNAs affected these pathways. In the AE group, nine differentially expressed miRNAs significantly influenced the PI3K-AKT signaling pathway, affecting 41 targets (*p* = 2.53 × 10^−17^, FDR: 1.44 × 10^−15^), and the FoxO signaling pathway, influencing 26 targets (*p* = 7.52 × 10^−17^, FDR: 3.66 × 10^−15^). These nine miRNAs play a role in the regulation of these pathways (Figure 9B). The three overlapping miRNAs between the AE and AE3 groups influenced the PI3K-AKT signaling pathway in second place, affecting 23 targets (*p =* 7.75 × 10^−14^, FDR: 9.86 × 10^−12^), and the FoxO signaling pathway in eighth place, influencing 14 possible targets (*p =* 1.63 × 10^−11^, FDR: 6.96 × 10^−10^) (Figure 9C).

**Figure 10:**
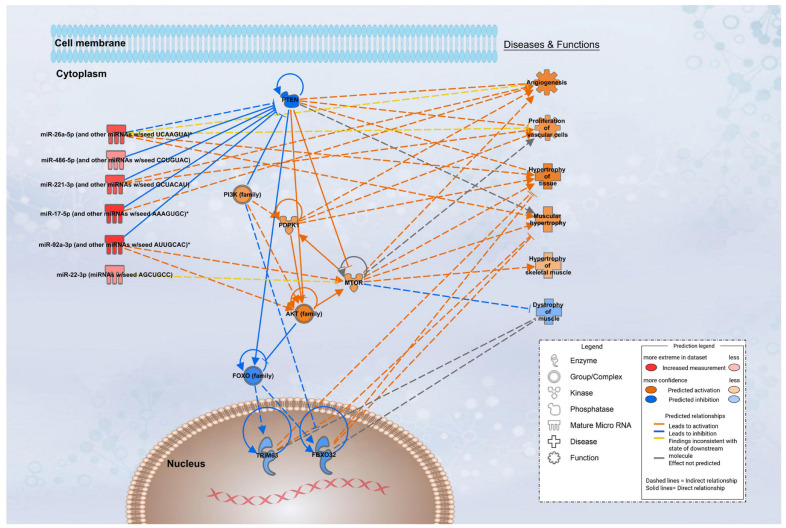
IPA network of differentially expressed miRNAs in the AE group. Upregulated miRNAs target PTEN, leading to predicted activation of the PI3K–AKT–mTOR pathway, inhibition of FOXO transcription factors, and suppression of MuRF1 (TRIM63) and Atrogin-1 (FBXO32). IPA predicts enhanced muscle hypertrophy and reduced atrophy, consistent with observed physiological adaptations. Asterisks (*) indicate duplicate identifiers mapped to a single molecule in the IPA global molecular network.

mRNAs most targeted by the differentially upregulated miRNAs in the AE group were also examined, finding that 10 miRNAs (hsa-miR-17-5p, hsa-miR-20a-5p, hsa-miR-22-3p, hsa-miR-25-3p, hsa-miR-26a-5p, hsa-miR-26b-5p, hsa-miR-92a-3p, hsa-miR-92b-3p, hsa-miR-221-3p, hsa-miR-486-5p) targeted the PTEN (phosphatase and tensin homolog) mRNA. Therefore, the 10 positively expressed miRNAs could potentially suppress PTEN expression, stimulating the PI3K-AKT pathway and inhibiting the FoxO pathway. IPA of the differentially expressed miRNAs further supported the involvement of anabolic signaling cascades. The miRNA Target Filter identified experimentally validated targets linking several upregulated miRNAs in the AE group (miR-17-5p, miR-20a-5p, miR-22-3p, miR-25-3p, miR-26a/b-5p, miR-92a/b-3p, miR-221-3p, and miR-486-5p) to PTEN, a negative regulator of PI3K/AKT signaling. Suppression of PTEN was predicted to enhance AKT activity, resulting in downstream activation of mTOR and stimulation of protein synthesis. Concomitantly, IPA predicted that increased AKT signaling would inhibit FOXO1/FOXO3 transcription factors, thereby reducing expression of the E3 ubiquitin ligases TRIM63 (MuRF1) and FBXO32 (Atrogin-1), which are central drivers of muscle proteolysis. Collectively, the network analysis predicted a shift toward an anabolic state characterized by activation of muscle hypertrophy and inhibition of muscle atrophy/dystrophy pathways, consistent with the physiological outcomes observed in the AE group. In addition, IPA indicated possible pro-angiogenic effects through PI3K/AKT-mediated vascular cell proliferation, suggesting broader systemic adaptations beyond skeletal muscle. The results are illustrated in Figure 10.

## 4. Discussion

To our knowledge, this is the first study investigating EV miRNAs in a young, resistance-trained population, combined with nutrient timing and chronic RE. We examined physiological changes and the role of EV-delivered miRNAs in regulating post-exercise adaptation, focusing on miRNA-mediated gene expression.

### 4.1. Body Composition

The AE group showed the greatest increase in skeletal muscle mass, the AE3 group showed a smaller but significant gain, and no change was observed in the CTRL group. These results, supported by effect size and post hoc power analysis, suggest that post-exercise dietary supplementation promoted hypertrophy in both AE and AE3, with the most pronounced effect of immediate consumption after exercise (AE). Body fat percentage decreased in all groups, indicating that muscle gains were due to nutrient timing combined with a structured training program rather than increased daily nutrient intake. While the placebo effect cannot be entirely ruled out, the obtained results indicate that for the average athlete, consuming a carbohydrate–protein complex immediately post-workout may be the most ideal strategy if the primary goal is muscle hypertrophy.

Only one previous study used a design similar to ours, in which immediate nutrient intake led to greater strength and muscle mass gains than delayed intake, with no change in body fat [40]. These results partly align with ours, underscoring the potential benefit of immediate protein supplementation, though unlike our AE3 findings, Esmarck et al. [40] observed a lean mass decrease with delayed intake. One study found greater lean mass gains and body fat changes with pre- and post-exercise supplementation than with morning/evening intake, concluding that nutrient timing can augment hypertrophy [41]. Their design differed from ours, as we did not include pre-exercise supplementation. Other studies using supplements without carbohydrates reported no significant performance or body composition changes between groups, although SM mass increased with training [42,43]. In these, timing included both pre- and post-exercise, while our study tested only post-exercise consumptions (AE, AE3) or none (CTRL). SM hypertrophy still occurred in AE and AE3 without a mass-gain diet, suggesting that training to exhaustion was sufficient for experienced subjects. Supplementation likely aided hypertrophy, especially when taken immediately post-exercise (AE). In the future, placebo-controlled studies are needed.

Our findings, consistent with prior research [44], indicate that nutrient timing may support hypertrophy, though total daily intake likely has a greater impact on post-exercise adaptations [45]. Immediate protein intake within one hour is not essential for an anabolic environment [46], as SM mass increased in both supplement groups, more so in AE. Nevertheless, immediate post-exercise ingestion of fast-absorbing maltodextrin and whey protein yielded greater strength, muscle growth, and reduced body fat than delayed or no supplementation. Nutrient timing may not surpass other dietary strategies, and further research should test this in both untrained and highly trained populations.

### 4.2. Muscle Strength Changes

Over 5 weeks, knee extensor strength increased in AE, showed an indicative change in AE3, and remained unchanged in CTRL, showing a similar trend to SM mass gains and reduced body fat, especially in AE. Previous studies likewise found greater strength gains with immediate post-exercise protein intake [40,41], though others reported no timing-related differences [42,43]. In our study, only AE showed a clear strength increase versus CTRL, suggesting nutrient timing may aid strength gains but with a smaller effect than previously thought. Immediate post-exercise supplementation is not essential for an anabolic response yet appears optimal for maximizing hypertrophy and strength.

### 4.3. Changes in EV Concentration and Size

Recent studies show that acute RE triggers EV release, with carried miRNAs displaying differential expression [27,47,48,49]. Intense acute RE increases vesicle concentration in men without altering vesicle size [50]. In our study, vesicle concentration rose significantly in AE, remained unchanged in AE3, and decreased in CTRL. Vesicle size was unchanged in AE and AE3 but decreased in CTRL. According to data, vesicle sizes fall within the small EV size range, indicating the successful isolation of small EVs. Our findings indicate that chronic RE was associated with changes in the profile of circulating EVs in plasma, which may be relevant to adaptations following physical activity. To elucidate mechanisms, analysis of bioactive cargo and surface protein/lipid signatures of small EVs is needed to determine their origin, destination, contents, and effects on target cells. Future studies should also assess the lipoprotein profile, as these overlap in size and density with EVs and may affect measurement accuracy [51]. Given the lack of a single optimal EV isolation method, our isolates may have included lipoproteins and protein aggregates [37,38]; however, protein digestion was performed prior to NTA measurements to minimize such contamination. Due to the differing baseline EV concentrations and the associated variability of the isolation method, we did not perform between-group statistical comparisons and instead focused our analysis on within-group changes.

In the AE group, appearing most anabolic, vesicle concentration increased, while it decreased in CTRL. In CTRL, SM mass was unchanged, but body fat decreased; reduced small EV levels may relate to reports that long-term exercise can lower certain EV subtypes, particularly in individuals with metabolic dysfunction [52]. Chronic RE has been shown to decrease some exosome markers, such as CD63, while others remain unchanged [22], and the lack of fluorescence-mode NTA here may have limited specificity. Only one prior study has examined chronic RE effects on the EV profile with validated isolation, finding no change in vesicle concentration or size but increased TSG101 expression [28], partly aligning with our results. Vesicle diameters matched between studies, but concentration differences likely reflect isolation and detection methods: precipitation yields more, but less pure EVs; SEC yields fewer, purer EVs [53]. In our work, high yield was prioritized for miRNA profiling from pooled samples.

### 4.4. Possible Involvement of Small EV-Delivered miRNAs in PI3K-AKT and FoxO Signaling Pathways

Most circulating miRNAs are packaged in EVs, whose phospholipid bilayer protects them from RNase degradation and enables intercellular communication [15,28,54,55,56,57,58]. However, whether circulating EV-miRNA concentrations are sufficient to induce biologically meaningful effects remains debated [59,60]. While most studies of EV-miRNAs and RE adaptation focus on acute effects [27,47,49,61,62], some address chronic responses as well [28]. In the present study, pooled EV samples were analyzed to profile 380 miRNAs across groups differing in anabolic status induced by RE and nutrient timing. The AE group showed predominantly upregulated miRNAs, whereas AE3 and CTRL exhibited mainly downregulated miRNAs.

FoxO activation increases Atrogin-1 and MuRF1 expression, driving protein breakdown [63], whereas AKT-mediated FoxO inhibition shifts protein balance toward hypertrophy [64]. PTEN negatively regulates PI3K-AKT-mTOR signaling, thereby limiting anabolic processes [65,66].

In our study, the AE group showed the most pronounced anabolic profile, with 10 miRNAs upregulated (hsa-miR-17-5p, -20a-5p, -22-3p, -25-3p, -26a-5p, -26b-5p, -92a-3p, -92b-3p, -221-3p, -486-5p) predicted to target PTEN, fostering an anabolic environment. Consistent with KEGG enrichment analysis, IPA identified PTEN as a central target of multiple upregulated miRNAs in AE, suggesting enhanced PI3K–AKT–mTOR signaling and reduced FOXO-driven transcription of catabolic genes. Although mRNA expression was not assessed, these miRNA changes are consistent with the observed increases in muscle mass and strength and reduced catabolic markers. Chronic RE studies often emphasize myomiRNAs, yet their circulating levels are typically low [27]. In line with this, only one myomiR (hsa-miR-486) was altered, potentially reflecting SM contribution; miR-486 inhibits FoxO1 and PTEN, reducing protein degradation and supporting hypertrophy [67,68]. Similar anabolic roles have been reported for miR-26a and miR-26b through PTEN–AKT–FOXO signaling in experimental muscle atrophy models [69,70,71]. Other miRNAs altered in AE (e.g., miR-20a, miR-22, miR-92a, miR-92b, and miR-221) have been linked to anabolic regulation, differentiation, or proliferation through pathways involving PTEN, AKT, or TGF-β signaling [72,73,74,75,76]. Kargl et al. reported sex-specific training-induced changes in EV miRNAs and increased AKT levels in muscle; the differences observed relative to our study may be attributable to variations in training protocols and methodologies [50].

Our findings are consistent with prior RE studies showing miRNA-dependent regulation of anabolic adaptations. Rivas et al. reported higher miR-92a levels in individuals exhibiting greater training-induced gains [23], while Xhuti et al. found increased miR-92a post-RE, especially in younger individuals [28]. The miR-17-92 cluster encodes six miRNAs (miR-17, -18a, -19a, -19b, -20a, -92a) involved in growth, differentiation, proteolysis, and survival [77,78]. In our study, miR-17, -20a, and -92a were upregulated in AE but downregulated in AE3 and CTRL. Prior work shows that these miRNAs can inhibit PTEN, with acute RE increasing miR-17-5p, -20a-5p, and -26b-5p in younger adults; hsa-miR-486-5p was linked to body composition changes [79]. miR-26b-5p shares a seed sequence with miR-26a-5p, which also changed in our study, reinforcing their potential involvement in AE-related anabolic regulation. While our findings provide insight into associations between RE, EV profiles, and their miRNA cargo, future functional studies are needed to directly verify the role of EV-derived miRNAs in metabolic regulation, including pathways such as PTEN and the PI3K–AKT–mTOR cascade.

### 4.5. Limitations

Our study has several limitations that future research should address. Understanding the possible placebo effect, incorporating placebo-controlled groups would be beneficial. Filling a nutrition diary can help with a more accurate evaluation of the results. Establishing more homogeneous groups would improve reliable intergroup comparisons. In addition to NTA measurements, lipid profile analysis could provide a more delicate structure of the expressed EVs; certain contaminant markers (e.g., APOA1 content of small EV preparations) were not assessed, although it would be important to exclude potential lipoprotein contamination, as recommended by the MISEV2023 guidelines [38]. Therefore, when interpreting the EV concentrations, it should be taken into account that the primary objective was to achieve the highest possible yield in order to enable the broadest possible miRNA profiling. NTA measurements were performed only in light scatter mode without the use of membrane-binding fluorescent tracers, which would be important to confirm the results and exclude non-lipid-based particles; future studies should address this aspect. In the present study, changes in EV concentration and the anabolic state should be interpreted as an association rather than a causal relationship, a distinction that future mechanistic research will need to clarify experimentally. Despite higher costs, using individual samples instead of pooled ones, along with high-throughput techniques, would be advantageous. While pooled samples reduce inter-sample variability, they may obscure specific characteristics of individual samples. RNA sequencing could further elucidate the full spectrum of miRNAs and other nucleic acids carried by EVs. A further limitation of the present study is that we did not perform molecular-level validation of the proposed EV-miRNA-mediated regulatory mechanisms. Although our analyses identified miRNAs and predicted signaling pathways potentially linking nutrient timing to skeletal muscle adaptation, we did not assess downstream targets such as PTEN protein expression, AKT/mTOR phosphorylation status, FOXO activity, or ubiquitination markers in muscle tissue or cell-based models. Future studies integrating such mechanistic experiments will be essential to directly confirm the functional impact of the identified EV-miRNAs on the PTEN and AKT-mTOR-FOXO signaling axis.

## 5. Conclusions

Our results demonstrate that RE combined with nutritional supplementation positively influenced muscle mass, knee extensor strength, and body composition, with the most pronounced effects observed in the AE group. While immediate post-exercise carbohydrate–protein intake appears beneficial for maximizing performance and hypertrophic responses, overall daily macronutrient intake remains a key determinant of long-term adaptations.

In addition, chronic RE was associated with distinct changes in EV-associated miRNA profiles, particularly in the AE group, where increased expression of miRNAs related to PTEN regulation was observed. These molecular adaptations may contribute to the establishment of a more favorable anabolic environment, supporting muscle growth and metabolic adaptations.

Despite these findings, the mechanistic role of EV-miRNAs in mediating exercise- and nutrition-induced adaptations remains to be fully elucidated. Future studies integrating targeted mechanistic approaches are needed to directly confirm the functional involvement of EV-miRNAs in the PTEN and AKT–mTOR–FOXO signaling pathways and to clarify how nutrient timing can be optimized to enhance recovery and long-term training adaptations.

## Figures and Tables

**Table 1:** Subject characteristics.

Groups	Age (years)	Body Weight (kg)	Height (cm)	Skeletal Muscle Mass (kg)	Body Fat Percentage (%)
AE (*n* = 7)	23 (±2.1)	85.5 (±7.6)	184.2 (±4.9)	38.8 (±2.4)	20 (±8)
AE3 (*n* = 7)	21.5 (±1.7)	79.7 (±9.2)	178.5 (±7.3)	37.5 (±2.7)	17 (±6.5)
CTRL (*n* = 6)	22.8 (±3.4)	83.7 (±9.4)	183.9 (±7.1)	40.3 (±3.8)	15.2 (±7.1)
All Groups (*n* = 20)	22.4 (±2.4)	82.9 (±8.6)	182.1 (±6.7)	38.8 (±3)	17.5 (±7.2)

Note. AE, consumed nutritional supplements immediately after exercise; AE3, consumed nutritional supplements three hours after exercise; CTRL, did not consume any nutritional supplements. Body composition was measured with an InBody 770 bio-impedance analyzer.

## Data Availability

The datasets generated and analyzed during the current study are available from the corresponding authors upon reasonable request due to time limitations.

## Supplementary Materials

The following supporting information can be downloaded at https://www.mdpi.com/article/10.3390/biomedicines14010127/s1, S1: Details of the training program; Table S1: Differentially expressed miRNAs and their RQ values.

## Abbreviations

The following abbreviations are used in this manuscript:

AE	consumed nutritional supplements immediately after exercise
AE3	consumed nutritional supplements three hours after exercise
CTRL	did not consume any nutritional supplements
EV	extracellular vesicle
FDR	false discovery rate
FoxO	forkhead box O
IPA	ingenuity pathway analysis
KEGG	Kyoto Encyclopedia of Genes and Genomes
miRNA	microRNA
MISEV2018	Minimal Information for Studies of Extracellular Vesicles 2018
NTA	nanoparticle tracking analysis
PFP	platelet-free plasma
PTEN	phosphatase and tensin homolog
RE	resistance exercise
SM	skeletal muscle
TEM	transmission electron microscopy
