# Supplementary material for: Chronic Resistance Exercise Combined with Nutrient Timing Enhances Skeletal Muscle Mass and Strength While Modulating Small Extracellular Vesicle miRNA Profiles
Source: Biomedicines. 2026 Jan 8;14(1):127. doi: 10.3390/biomedicines14010127 (PMC12839061; doi:10.3390/biomedicines14010127)
Supplement: Supplementary file 1 [file biomedicines-14-00127-s001.zip › Supplementary Material S1.pdf]

Hypertrophy-Specific Training Plan for 5 Weeks

First week
Monday: Chest & Abs
- **Bench Press** – 4x8 *(Rest: 60-90 sec)*
- **Machine Flyes** – 4x10 *(Rest: 30-45 sec)*
- **45-Degree Dumbbell Presses** – 4x8 *(Rest: 60-90 sec)*
- **Tricep Dip Machine (Leaning Forward)** – 4x8 *(Rest: 45-60 sec)*
- **Cable Flyes** – 4x10 *(Rest: 30-45 sec)*
- **Sit-ups on the Roman Chair** – 4x15-20 *(Rest: 30-45 sec)*

Tuesday: Leg Day
- **Leg Extensions** – 4x8 *(Rest: 60-90 sec)*
- **Leg Curls** – 4x8 *(Rest: 60-90 sec)*
- **Free Weight Squats** – 4x8 *(Rest: 90-120 sec)*
- **Leg Presses** – 4x8 *(Rest: 60-90 sec)*
- **Romanian Deadlift** – 4x8 *(Rest: 90-120 sec)*
- **Standing Calf Raises** – 4x15 *(Rest: 30-45 sec)*

Thursday: Back & Abs
- **Wide Grip Pulldown** – 4x8 *(Rest: 60-90 sec)*
- **Barbell Row** – 4x8 *(Rest: 90-120 sec)*
- **Narrow Grip Pulldown** – 4x8 *(Rest: 60-90 sec)*
- **Machine Row** – 4x8 *(Rest: 60-90 sec)*
- **Stiff Arm Lat Pulldown** – 4x12 *(Rest: 45-60 sec)*
- **Leg Raises** – 4x10-15 *(Rest: 30-45 sec)*

Friday: Shoulders & Arms
- **Shoulder Presses with Dumbbells** – 4x8 *(Rest: 60-90 sec)*
- **Lateral Raises** – 4x8 *(Rest: 45-60 sec)*
- **Rear Delt Fly in the Machine** – 4x8 *(Rest: 45-60 sec)*
- **French Curl (EZ Bar Bicep Curl)** – 4x8 *(Rest: 60-90 sec)*
- **Hammer Curl** – 4x8 *(Rest: 60-90 sec)*
- **Skull Crushers (EZ Bar Triceps Extension)** – 4x8 *(Rest: 60-90 sec)*
- **Triceps Pushdown (Cable)** – 4x8 *(Rest: 45-60 sec)*

Second Week
Monday: Chest & Abs
- **45-Degree Bench Press** – 2x8, 2x6 *(Rest: 60-90 sec)*
- **Pull Over** – 4x10 *(Rest: 45-60 sec)*
- **Dumbbell Presses** – 2x8, 4x6 *(Rest: 60-90 sec)*
- **Push Down in the Tricep Dip Machine (Leaning Forward)** – 4x6 *(Rest: 60-90 sec)*
- **Cable Flyes** – 4x10 *(Rest: 45-60 sec)*
- **Sit-ups on the Roman Chair** – 4x15-20 *(Rest: 30-45 sec)*

Tuesday: Leg Day
- **Squats** – 2x8, 2x6 *(Rest: 90-120 sec)*
- **Leg Presses** – 2x8, 2x6 *(Rest: 90-120 sec)*
- **Romanian Deadlift** – 4x8 *(Rest: 90-120 sec)*
- **Leg Extension** – 4x6 *(Rest: 60-90 sec)*
- **Leg Curl** – 4x6 *(Rest: 60-90 sec)*
- **Seated Calf Raises** – 4x15 *(Rest: 30-45 sec)*

Thursday: Back & Abs
- **Pull-ups (Machine or Free Weight)** – 2x8, 2x6 *(Rest: 90-120 sec)*
- **Deadlift** – 2x8, 2x6 *(Rest: 120-150 sec)*
- **Reverse Grip Pulldown** – 2x8, 2x6 *(Rest: 60-90 sec)*
- **T-Bar Row** – 4x6 *(Rest: 90-120 sec)*
- **Stiff Arm Push Down** – 4x12 *(Rest: 45-60 sec)*
- **Leg Raises** – 4x10-15 *(Rest: 30-45 sec)*

Friday: Shoulders & Arms
- **Military Press** – 2x8, 2x6 *(Rest: 60-90 sec)*
- **Lateral Raises** – 2x8, 2x6 *(Rest: 45-60 sec)*
- **Face Pull** – 4x8 *(Rest: 45-60 sec)*

- **Barbell Curl** – 2x8, 2x6 *(Rest: 60-90 sec)*
- **Dumbbell Curl** – 2x8, 2x6 *(Rest: 60-90 sec)*
- **Skull Crushers with Barbell** – 2x8, 2x6 *(Rest: 60-90 sec)*
- **Cable Push Down with Rope** – 2x8, 2x6 *(Rest: 60-90 sec)*

Third week

Monday: Chest & Abs

- **Bench Press** – 4x6 *(Rest: 60-90 sec)*
- **Dumbbell Flyes** – 4x12 *(Rest: 45-60 sec)*
- **45-Degree Dumbbell Press** – 4x6 *(Rest: 60-90 sec)*
- **Pull Over** – 4x12 *(Rest: 45-60 sec)*
- **Machine Flyes** – 4x10 *(Rest: 45-60 sec)*
- **Sit-ups in the Machine** – 4x15-20 *(Rest: 30-45 sec)*

Tuesday: Leg Day

- **Smith Squats** – 4x6 *(Rest: 90-120 sec)*
- **Hack Squats** – 4x6 *(Rest: 90-120 sec)*
- **Lying Hamstring Curls** – 4x8-10 *(Rest: 60-90 sec)*
- **Romanian Deadlift with Dumbbells** – 4x8-10 *(Rest: 90-120 sec)*
- **Leg Press** – 4x15 *(Rest: 60-90 sec)*
- **Standing Calf Raises** – 4x15-20 *(Rest: 30-45 sec)*

Thursday: Back & Abs

- **Wide Grip Pulldown** – 4x6 *(Rest: 60-90 sec)*
- **Deadlift** – 4x6 *(Rest: 120-150 sec)*
- **Narrow Grip Pulldown** – 4x8-10 *(Rest: 60-90 sec)*
- **T-Bar Rows** – 4x6 *(Rest: 90-120 sec)*
- **Stiff Arm Push Down** – 4x12 *(Rest: 45-60 sec)*
- **Crunches** – 4x Max Reps *(Rest: 30-45 sec)*

Friday: Shoulders & Arms

- **Shoulder Press in a Machine** – 4x6 *(Rest: 60-90 sec)*
- **Lateral Raises** – 4x6-10 *(Rest: 45-60 sec)*
- **Rear Shoulder Machine** – 4x6-10 *(Rest: 45-60 sec)*
- **EZ Bar Bicep Curls** – 4x6 *(Rest: 60-90 sec)*
- **Scott Bicep Curls (Free Weight)** – 4x6-8 *(Rest: 60-90 sec)*
- **EZ Bar Skull Crushers** – 4x6 *(Rest: 60-90 sec)*
- **Reverse Grip Tricep Pushdowns** – 4x10 *(Rest: 45-60 sec)*

Fourth Week

Monday: Chest & Abs

- **Dumbbell Presses** – 4x10-12 *(Rest: 60-90 sec)*
- **Dumbbell Flyes** – 4x12-15 *(Rest: 45-60 sec)*
- **45-Degree Bench Press in Smith Machine** – 4x10-12 *(Rest: 60-90 sec)*
- **Pull Over** – 4x12-15 *(Rest: 45-60 sec)*
- **Tricep Dip Machine** – 4x12 *(Rest: 60-90 sec)*
- **Cable Flyes** – 4x12 *(Rest: 45-60 sec)*
- **Leg Raises** – 4x Max Reps *(Rest: 30-45 sec)*

Tuesday: Leg Day

- **Lying Leg Curl** – 4x15 *(Rest: 60-90 sec)*
- **Leg Extension** – 4x15 *(Rest: 60-90 sec)*
- **Squats with Free Weight** – 4x15 *(Rest: 90-120 sec)*
- **Leg Presses** – 4x15-20 *(Rest: 60-90 sec)*
- **Romanian Deadlift with Dumbbells** – 4x15 *(Rest: 90-120 sec)*
- **Standing Calf Raises** – 4x15-20 *(Rest: 30-45 sec)*
- **Seated Calf Raises** – 4x15-20 *(Rest: 30-45 sec)*

Thursday: Back & Abs

- **Reverse Wide Grip Pulldown** – 4x10-12 *(Rest: 60-90 sec)*
- **Narrow Grip Pulldown** – 4x10-12 *(Rest: 60-90 sec)*
- **Rack Deadlift** – 4x10-12 *(Rest: 90-120 sec)*
- **Dumbbell Rows** – 4x10-12 *(Rest: 60-90 sec)*
- **Machine Row** – 4x10-12 *(Rest: 60-90 sec)*

- **Sit-ups** – 4x Max Reps *(Rest: 30-45 sec)*

Friday: Shoulders & Arms

- **Lateral Raises** – 4x10-15 *(Rest: 45-60 sec)*
- **Face Pull** – 4x10-15 *(Rest: 45-60 sec)*
- **Shoulder Presses with Dumbbells** – 4x10-15 *(Rest: 60-90 sec)*
- **Cable Arm Curls with Straight Bar** – 4x10-15 *(Rest: 45-60 sec)*
- **Hammer Curls** – 4x10-15 *(Rest: 45-60 sec)*
- **Triceps Pushdown with Rope** – 4x10-15 *(Rest: 45-60 sec)*
- **Kickback** – 4x10-15 *(Rest: 45-60 sec)*

Fifth week

Monday: Chest & Abs

- **Bench Press** – 4x6 *(Rest: 60-90 sec)*
- **45-Degree Dumbbell Flyes** – 4x10 *(Rest: 45-60 sec)*
- **45-Degree Dumbbell Press** – 4x6 *(Rest: 60-90 sec)*
- **Pull Over** – 4x10 *(Rest: 45-60 sec)*
- **Tricep Dips Machine** – 4x6 *(Rest: 60-90 sec)*
- **Cable Flyes** – 4x12 *(Rest: 45-60 sec)*
- **Leg Raises** – 4x Max Reps *(Rest: 30-45 sec)*

Tuesday: Leg Day

- **Squats with Free Weight** – 4x6 *(Rest: 90-120 sec)*
- **Leg Presses** – 4x8 *(Rest: 90-120 sec)*
- **Lying Hamstring Curls** – 4x8 *(Rest: 60-90 sec)*
- **Romanian Deadlift** – 4x8 *(Rest: 90-120 sec)*
- **Lunges** – 4x8 *(Rest: 60-90 sec)*
- **Standing Calf Raises** – 4x15-20 *(Rest: 30-45 sec)*
- **Seated Calf Raises** – 4x15-20 *(Rest: 30-45 sec)*

Thursday: Back & Abs

- **Pull-Ups** – 4x6-8 *(Rest: 90-120 sec)*
- **Deadlift** – 4x6 *(Rest: 120-150 sec)*
- **Narrow Grip Pulldown** – 4x6-8 *(Rest: 60-90 sec)*
- **T-Bar Row** – 4x6-8 *(Rest: 90-120 sec)*
- **Machine Row** – 4x6-8 *(Rest: 60-90 sec)*
- **Stiff Arm Push Down** – 4x10 *(Rest: 45-60 sec)*
- **Sit-ups** – 4x Max Reps *(Rest: 30-45 sec)*

Friday: Shoulders & Arms

- **Military Press** – 4x6 *(Rest: 60-90 sec)*
- **Rear Shoulder Machine** – 4x8 *(Rest: 45-60 sec)*
- **Overhead Presses with Dumbbells** – 4x6 *(Rest: 60-90 sec)*
- **Lateral Raises** – 4x8 *(Rest: 45-60 sec)*
- **EZ Bar Arm Curls** – 4x6-8 *(Rest: 60-90 sec)*
- **Hammer Curls** – 4x6-8 *(Rest: 60-90 sec)*
- **EZ Bar Skull Crushers** – 4x6-8 *(Rest: 60-90 sec)*
- **Tricep Extension** – 4x8 *(Rest: 60-90 sec)*
